# DataTri, a database of American triatomine species occurrence

**DOI:** 10.1038/sdata.2018.71

**Published:** 2018-04-24

**Authors:** Soledad Ceccarelli, Agustín Balsalobre, Paula Medone, María Eugenia Cano, Rodrigo Gurgel Gonçalves, Dora Feliciangeli, Darío Vezzani, Cristina Wisnivesky-Colli, David E Gorla, Gerardo A Marti, Jorge E Rabinovich

**Affiliations:** 1 Centro de Estudios Parasitológicos y de Vectores (CEPAVE-CCT-La Plata-CONICET-UNLP), La Plata, Buenos Aires 1900, Argentina; 2 Secretaría de Educación, Colima, Colima 28000, México; 3 Laboratório de Parasitologia Médica e Biologia de Vetores, Faculdade de Medicina, Universidade de Brasília, Distrito Federal 70040, Brasil; 4 Instituto Multidisciplinario sobre Ecosistemas y Desarrollo Sustentable, Facultad de Ciencias Exactas, Universidad del Centro de la Provincia de Buenos Aires-CONICET, Tandil, Buenos Aires 7000, Argentina; 5 Departamento de Ecología, Genética y Evolución, UBA, Capital Federal, Buenos Aires 1428EGA, Argentina; 6 Instituto de Altos Estudios Espaciales Mario Gulich (CONAE_Universidad Nacional de Córdoba-CONICET), Falda de Cañete, Córdoba 5187, Argentina

**Keywords:** Entomology, Ecological epidemiology, Parasitic infection

## Abstract

*Trypanosoma cruzi*, the causative agent of Chagas disease, is transmitted to mammals - including humans - by insect vectors of the subfamily Triatominae. We present the results of a compilation of triatomine occurrence and complementary ecological data that represents the most complete, integrated and updated database (*DataTri)* available on triatomine species at a continental scale. This database was assembled by collecting the records of triatomine species published from 1904 to 2017, spanning all American countries with triatomine presence. A total of 21815 georeferenced records were obtained from published literature, personal fieldwork and data provided by colleagues. The data compiled includes 24 American countries, 14 genera and 135 species. From a taxonomic perspective, 67.33% of the records correspond to the genus *Triatoma*, 20.81% to *Panstrongylus*, 9.01% to *Rhodnius* and the remaining 2.85% are distributed among the other 11 triatomine genera. We encourage using *DataTri* information in various areas, especially to improve knowledge of the geographical distribution of triatomine species and its variations in time.

## Background & Summary

Chagas disease, caused by the protozoan *Trypanosoma cruzi*, is transmitted mainly by triatomine (Hemiptera: Reduviidae) insect vectors (through their feces), but may also be transmitted from mother to child, by blood transfusions or some organ transplants, and through oral transmission. These multiple routes of transmission make Chagas disease an important public health problem, primarily in the Americas but also in other continents^1^. A compilation of geographic and ecological information about triatomines is considered to be particularly important, given that these insect vectors are one of the main routes of *T. cruzi* transmission and no complete and integrated database is available on triatomine occurrences. Such information can be used to carry out actions and support programmes for disease prevention and vector control, by public health agencies.

One of the last compilations of American triatomine species was the publication by Carcavallo *et al.*^2^, bringing up to date the geographic and altitudinal distribution of 115 American triatomine species known at that time. Although this material is still very useful, as the number of species has increased and there have been several changes in some taxonomic classifications^3^, this information needed to be updated. Furthermore, from a methodological perspective, the geographic distributions in Carcavallo *et al.*^2^ were presented as range maps based on unspecified methodologies and mostly "gray" bibliography. Triatomine species occurrences used to make these range maps were not georeferenced, and the geographic information relied primarily on political boundaries (provinces or departments, and even an entire country), with scarce mention of specific localities.

Since 1998, several regional and smaller scale compilations on the geographic distribution of triatomines have been published. Some of them analyze and describe the geographic distributions of species from a single country such as Brazil^4,5^, Colombia^6^ and Mexico^7,8^; others describe lists of species, such as those published for French Guiana^9^, Suriname^10^, Peru^11^ and Venezuela^12^, or provide lists of valid species (checklists) for all triatomine species^13^, including systematic updates. Despite these valuable publications, a database with integrated information on the georeferenced occurrence of all -or at least the majority- American triatomine species, is still unavailable.

**Figure 1:**
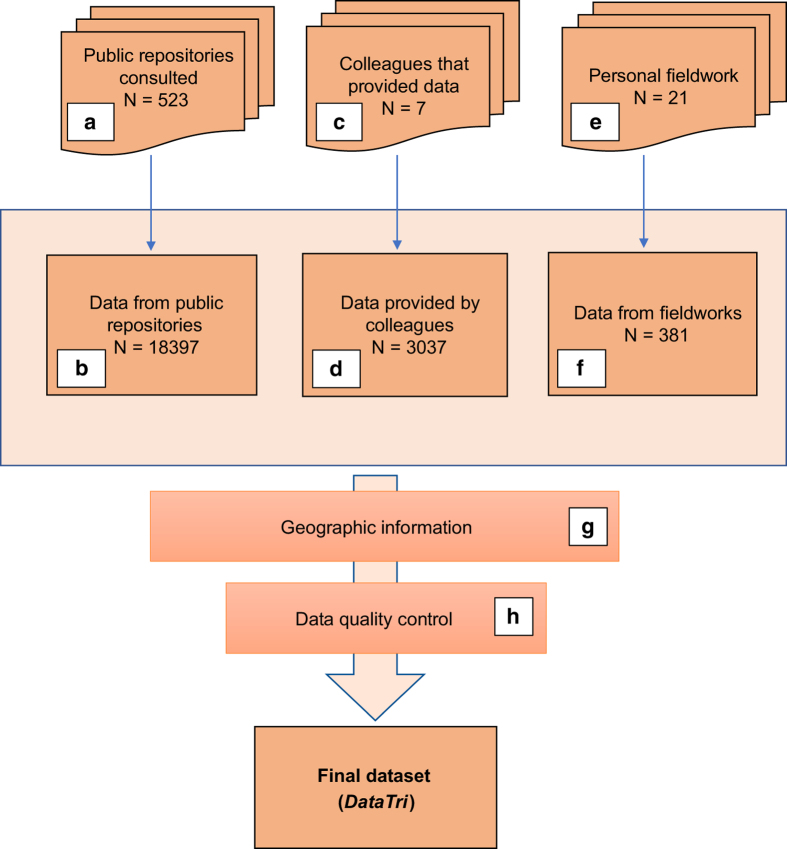
Schematic overview of data compilation, including information sources used and data compilation processes. *N*=number of records. Steps a-g are described in the Methods section. Step h is described in the Technical Validation section.

The main goal of this work is to provide a public geodatabase with updated and well referenced occurrence data for triatomine species in the Americas. This work is the result of an exhaustive as possible review of public information combined with substantial interinstitutional collaboration (Fig. 1), which integrated not only geographical but also ecological data for 135 American triatomine species from 24 American countries. This geodatabase, hereinafter called *DataTri*, may contribute not only to improve the knowledge of geographical distributions of every triatomine species but also to design improved strategies for health promotion and vectorial control. We believe it will be of practical use for both the academic and educational community, as well as for those institutions responsible for Public Health prevention, promotion and vectorial control activities. *DataTri* may be the first database that includes accurate georeferenced information for most of the known records of American triatomine species.

## Methods

### Description of *DataTri* fields

We compiled most of the useful available information associated to each triatomine species and attached the data to each *DataTri* field, be it characteristics of the specimens collected or of the sampled sites. The 19 fields used to systematize the information were included in the following 10 categories: 1) systematic (*scientificName* and *taxonRemarks*), 2) administrative divisions (*country*, *stateProvince, municipality* and *locality*), 3) geographical coordinates (*decimalLatitude*, *decimalLongitude* and *georeferenceSources*; see details in the “Data georeferencing process” section below), 4) specimens collection date (*year*, *month* and *day*), 5) name/s of specimen collector/s (*recordedBy*), 6) sampled habitat (*habitat*), 7) sampling protocol (*samplingProtocol)*, 8) total number of individuals sampled (*individualCount*), 9) reference of the record (*associatedReferences*), and 10) data source type *(Data and Data II)*. These 19 independent fields are part of the data file mentioned in the Data Records section.

The following sections provide some details about some of the above-mentioned fields, which require specific clarifications.

### Systematic fields

When appropriate, the *taxonRemarks* field included notes and/or references about synonyms or revalidations of the species described in the corresponding record.

### Administrative division fields

*Locality* field refers to the site nearest to the geographic coordinates, not necessarily the name of the locality where the specimens were collected.

### Specimens collection date fields

When a group of specimens or habitat information corresponded to a certain period of time but with specific dates identified, the data was split into different records. If this splitting was not possible, each record included the original time interval information (years, month or days) (e.g. “specimens were collected between 2005–2006”).

### Habitat sampled field

The *habitat* field reflects in what kind of habitat the triatomines were collected, and they were classified into three categories: domicile, peridomicile and sylvatic. When specific habitat information was aggregated, the habitat was expressed as a combination of those three categories (e.g., domicile-peridomicile, domicile-sylvatic, peridomicile-sylvatic or domicile-peridomicile-sylvatic).

### Sampling protocol field

Sampling protocols were classified into three categories: i) *active search* when the searching involved specialized staff, ii) *community participation* when the searching involved community help, and iii) *passive collection* when different types of traps (e.g. Light or Noireau traps) were used.

### Reference of the record field

The reference of each record is the source of information: either a colleague or an institution that provided the data, or the bibliographical reference to a published article.

### Data source type fields

The *Data* and *Data II* fields refer to the information source whence data were compiled (public repositories, data provided by colleagues or personal fieldwork). In some cases, data for a record was obtained from a published article, but the geographic coordinates were provided by the authors of the publication through personal communication. In those cases, the *Data II* field includes the name of the colleague that provided the geographic coordinates.

### Information source types and compilation of triatomine species data

Information source types were identified and selected for data compilation, following the procedure described in Fig. 1. To build the final dataset, data for each triatomine species were obtained by carrying out a detailed and exhaustive as possible review of information. No specific temporal range limits were set, to obtain the greatest possible amount of historical data from as many American countries as possible. Regarding to published information, several types of public bibliographic repositories available online (BioOne, Google Scholar, PLoS, PubMed, Scielo, ScienceDirect, Wiley) were reviewed using terms such as “Chagas disease”, “Triatominae”, “*Trypanosoma cruzi*”, without language restriction. We also reviewed the public and open access triatomine bibliographic database called *BibTri* (http://bibtri.com.ar), and some triatomine-specific reference books or monographs on Chagas disease vectors^2,13–15^. In the case of public data repositories, several open-data or institutional portals such as SpeciesLink (http://www.splink.org.br/), GBIF (https://www.gbif.org/), BoldSystems (http://www.boldsystems.org/index.php) and UNAM (http://www.ib.unam.mx/) were reviewed (step A). The results were then integrated as “data from public repositories” (step B).

When published articles mentioned unpublished datasets, the authors were contacted and asked to provide geographic coordinates or at least localities data to georeference them (step C). Geographic information provided by colleagues upon request was compiled as “data provided by colleagues” (step D).

Data obtained from fieldwork done in Argentina and Bolivia collected by active searching (manual collection) and/or passive collection methods (light traps and baited traps^16,17^) performed by members of the triatomine laboratory of the “Centro de Estudios Parasitológicos y de Vectores” (CEPAVE-CCT La Plata CONICET-UNLP) (step E), were integrated as “data from fieldwork” (step F). Data compiled from the three data sources were combined to build a preliminary dataset.

### Data georeferencing process

To rigorously associate each record to a specific location in the geographical space, the data must have information expressed in geographic coordinates (latitude and longitude). If no geographic coordinates were available, the site name was used together with information on administrative divisions to attain an accurate location using gazetteers (Global Gazetteer Fallingrain, version 2.2, http://www.fallingrain.com/ or Google Earth, https://www.google.com.ar/intl/es/earth/). If the geographic coordinates were not expressed in decimal degrees, they were converted using a coordinate conversion application (http://www.maclasa.com/coordenadas/). When possible, the geographic coordinates were verified in both gazetteers. When only the geographic coordinates were available, the corresponding administrative divisions were completed using a Species Link tool called GeoLoc (http://splink.cria.org.br). The datum (coordinate system and set of reference points used to locate places on Earth) used for all geographic records was WGS84 (World Geodetic System 1984) (step G).

The final dataset was built after a data quality control (step H) (see Technical Validation section).

## Data Records

The full workflow and number of individual data that contributed to the final dataset is given in Fig. 1. A total of 21815 data records were compiled and entered into a data file, which is stored in *figshare* (Data Citation 1). The individual data file within Data Citation 1 is the result of the compilation of all valid occurrences of American triatomines. Each individual record contains data related to spatial, temporal and complementary ecological information, as described in the previous Section.

The elements reviewed from public information sources were articles from scientific journals, databases from websites, museums or other institutions’ collections hosted in websites, institutional reports or bulletins, abstracts from scientific meetings (congresses, workshops, etc.) and PhD theses. In total, 84.3% of the records were obtained from public repositories, 14% from data provided by colleagues and 1.7% from personal fieldwork (Table 1).

**Figure 2:**
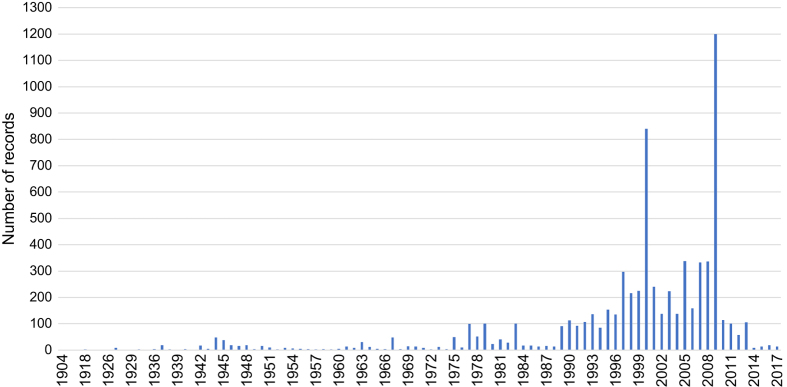
Frequency distribution of the number of records per year.

The temporal range covered in *DataTri* is from 1904 to 2017. Date information was available for 65% of the records and 74% of them comprise data only from the last 30 years (Fig. 2).

**Figure 3:**
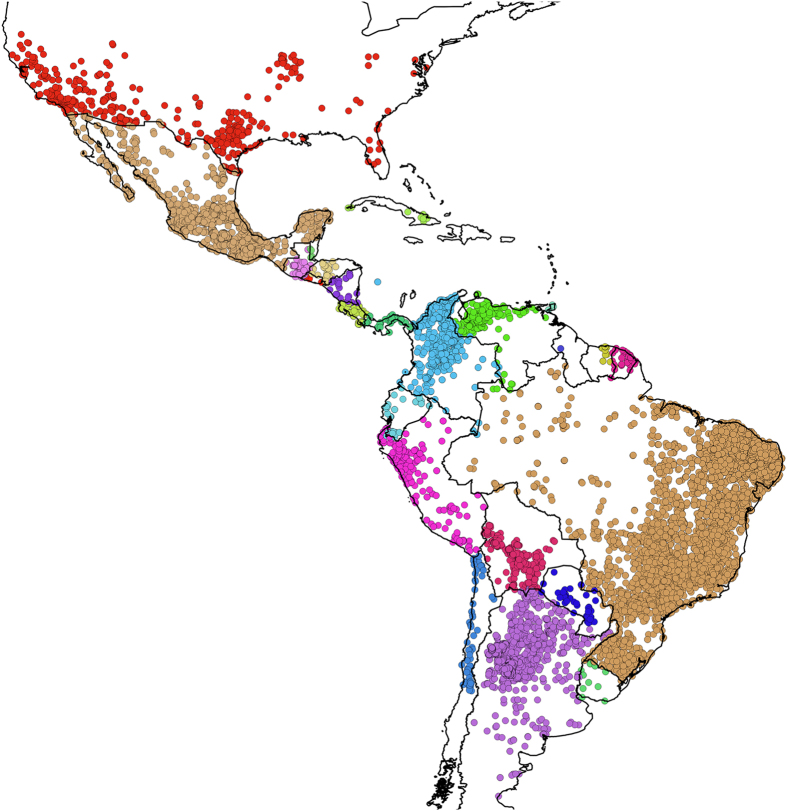
Geographic distribution of triatomine occurrence data. Each color represents the dataset of all species in *DataTri* from each country.

The geographical coverage of *DataTri* includes a wide range, from the United States of America to the southern part of Argentina and Chile. This range coincides with the southernmost and northernmost records of triatomine geographical distribution known to date. The data compiled span 24 American countries (Fig. 3), 14 genera and 135 of the 150 extant species^18^ (see Supplementary File 1 for more details). From a taxonomic perspective, 67.33% of the records correspond to the genus *Triatoma*, 20.81% to *Panstrongylus*, 9.01% to *Rhodnius* and the remaining 2.85% are distributed among the other 11 triatomine genera (Table 2).

**Figure 4:**
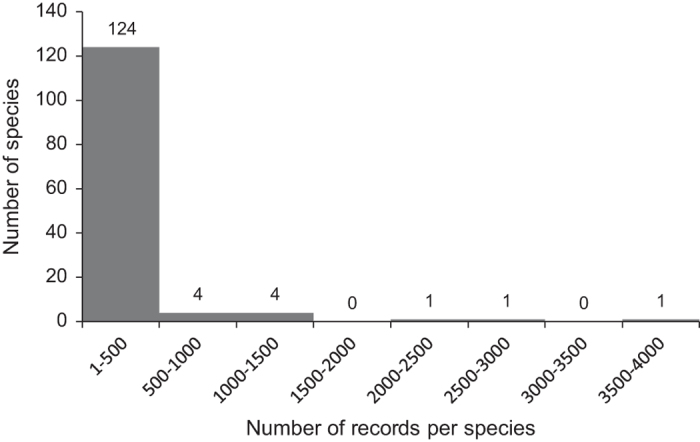
Frequency distribution of triatomine species amount per number of records. Numbers of species represented by each interval is indicated above each column.

The number of records per species ranges from one to 3881 records, with 124 triatomine species (92%) having between one and 500 records (Fig. 4). The other 11 species have more than 500 records, with *T. dimidiata*, *P. megistus* and *T. infestans* being the species with most occurrence data (2121, 2726 and 3881 records respectively).

The number of records by country ranges from a single datum for Guyana, to almost 8912 records for Brazil (Table 3). Of these records, 56% included information on habitat type and were grouped into two habitat categories: domicile-peridomicile (49%) and sylvatic (7%) (Table 3). The number of triatomine species by country included in *DataTri* is given in Table 3.

## Technical Validation

The dataset was subjected to an exhaustive quality control. First, each datum was extracted by one person and checked by two other people to ensure accuracy and to verify that records were not duplicated. Subsequently, data were checked to avoid any kind of error (e.g. typing, georreferencing, incorrect locations, taxonomic synonyms, errors in spelling of administrative divisions, etc.) that might have arisen during compilation or data entry. To correct and remove typographical errors and spelling mistakes in the names of administrative divisions, we used the *OpenRefine* software (http://openrefine.org/) that aids in the detection of this type of errors in large datasets.

All geographic coordinates were checked using open GIS software (QGIS and DivaGIS) to detect georeferenced errors and incorrect locations, ensuring that each point corresponded to a location on the continent and in the correct country. Any outlier coordinates that were geographically distant from the known distribution of a given species were investigated to ensure that they were correct. During the validation of geographic coordinates (Specieslink), it was detected that some occurrence data from public sources were located outside the continent or within continental waterbodies. These data may have been erroneously georeferenced by the authors of the original scientific publication; however, if we considered these data were sufficiently valuable to be kept, we decided to carry out the following procedure: if the *Country, Stateprovince*, *Municipality* and *Locality* fields were provided by the authors, we assigned the correct geographic coordinate, as is explained in the Data georreferencing process section, taking as a reference the name of the locality contributed by the authors.

To detect taxonomic synonym errors, we used the most recent triatomine checklist of currently valid species^13^. If any species name was suspected to be outdated because the synonyms were established after publication of the most recent triatomine species checklist, we consulted updated bibliography or requested the expert opinion of other colleagues.

## Usage Notes

As *DataTri* information has been collected using different procedures, this compilation may contain some inherent biases that should be addressed when the data are intended to be used.

Most of the data were obtained from papers published in scientific journals, accompanied by those provided by colleagues. Although data span 24 countries, there were some countries such as Brazil, Mexico and Argentina for which the volume of data was higher than for the rest. In the case of Mexico and Brazil the number of occurrence data per country included in *DataTri* seems to be mainly influenced by two factors: (i) the number of triatomine species present in each country (both countries have the highest number of triatomine species), and (ii) by the number of occurrence data published and provided by colleagues, (also Mexico and Brazil are the countries with the largest amount of data collected); an explanation for the latter factor goes beyond the goal of this paper. In the case of Argentina, there is also a large number of occurrence data, but in this case, this is because the *DataTri* initiative arose from Argentinian researchers with a great occurrence data contribution. With regards to habitat sampling we recognize that there is a potential bias in favor of the domiciliar and peridomiciliar habitats because those are the habitats of major epidemiological importance and the target of the vector control campaigns. Additionally, the paucity of sylvatic habitat data also results from the difficulty of sampling procedures in the large variety of sylvatic habitats used by the triatomines. Finally, it should also be clarified that the date information is not available in 35% of the records, thus, we recommend that any analysis based on this dataset should use methods that take such biases into account.

Despite the information biases described above, *DataTri* constitutes a valuable compilation of American triatomines geographic data that is as complete, updated and integrated as possible. Currently, compared to other public biodiversity databases, *DataTri* triples the number of records of triatomine data found in the GBIF database, and its volume is even higher when compared with other public databases such as BISON (https://bison.usgs.gov/#home), INaturalist (https://www.inaturalist.org/) or Museums websites. Thus, *DataTri* has a better data representativeness regarding to the number of species, the number of countries and that each record has a location with an accurate geographic coordinate.

An accurate spatial information based upon geographic coordinates also allows to link and complement with other databases such as VectorBase, that provides data on vector genetic information, and another dataset published in Scientific Data^19^ that provides data on *Trypanosoma cruzi* occurrence/prevalence in humans, alternative hosts and triatomines. In addition, as this dataset is hosted in an open and public repository, we hope that it will contribute to fulfill national and international goals such as promoting the exchange of biological information, increasing and improving accessibility of such information, providing biological data produced and compiled in several countries, and enhancing knowledge of both the biodiversity and epidemiological data related to Chagas disease.

## Additional information

**How to cite this article**: Ceccarelli S. *et al.* DataTri, a database of American triatomine species occurrence. *Sci. Data* 5:180071 doi: 10.1038/sdata.2018.71 (2018).

**Publisher’s note:** Springer Nature remains neutral with regard to jurisdictional claims in published maps and institutional affiliations.

## Supplementary Material



Supplementary Information

## Figures and Tables

**Table 1:** Types of information sources reviewed for data compilation and used to build *DataTri*.

**Type of information source**	**Number of elements reviewed**	**Number of records compiled**	**% of data contribution**
Public repositories	523	18397	84.3
Data provided by Colleagues	7	3037	14
Personal fieldwork	21	381	1.7
The chart presents the number of elements corresponding to each information source type, number of records compiled from each information source type and percentage of contribution from each information source.			

**Table 2:** American Triatominae genera, number of species per genus, number of records and percentage of data contribution per genus represented in *DataTri*.

**Genus**	**N° of species of each genus**	**N° de records of each genus**	**%**
*Triatoma*	73	14672	67.26
*Panstrongylus*	14	4548	20.81
*Rhodnius*	21	1970	9.01
*Psammolestes*	3	252	1.15
*Eratyrus*	2	151	0.69
*Paratriatoma*	1	58	0.27
*Mepraia*	3	53	0.24
*Cavernicola*	2	42	0.19
*Belminus*	8	24	0.11
*Microtriatoma*	2	17	0.08
*Dipetalogaster*	1	15	0.07
*Alberprosenia*	2	4	0.02
*Hermanlentia*	1	4	0.02
*Parabelminus*	2	3	0.01
TOTAL	135	21813	100

**Table 3:** Number of triatomine species included in *DataTri*, number of total records and records per habitat type by country.

**Country**	**Number of Species**	**Number of records**	**Number of records classified as “Domicile-Peridomicile habitat”**	**Number of records classified as “Sylvatic habitat”**
Brazil	68	8912	6874	368
Mexico	29	3866	71	17
Colombia	27	982	74	20
Venezuela	18	793	322	21
Peru	17	290	175	76
Argentina	16	3893	2661	490
Ecuador	13	75	55	0
Bolivia	11	653	273	199
French Guyana	11	93	0	93
United States of America	11	759	0	2
Panama	8	105	56	1
Nicaragua	7	44	8	0
Paraguay	7	118	52	51
Costa Rica	5	210	11	3
Uruguay	5	17	6	9
Chile	4	104	29	39
Guatemala	4	794	59	10
Surinam	4	31	0	0
Honduras	3	47	7	0
Trinidad and Tobago	3	8	1	4
Cuba	2	8	2	1
El Salvador	2	5	4	0
Belize	1	7	6	1
Guyana	1	1	0	0
The information is ordered by number of species per country.				
