# Supplementary material for: DataTri, a database of American triatomine species occurrence
Source: Sci Data. 2018 Apr 24;5:180071. doi: 10.1038/sdata.2018.71 (PMC5914284; doi:10.1038/sdata.2018.71)
Supplement: Supplementary Information [file sdata201871-s2.pdf]

Supplementary Material

Table 1. Systematics of the 150 extant species and the two fossils currently described according to the classification of Justi & Galvao[1]. The chart includes taxonomic and geographical information. The "x" indicates the 135 species present in *DataTri*.

Tribu	Triatomine species	Species included in *DataTri*	Continent	Author/s
Alberproseniini	*Alberprosenia goyovargasi*	x	America	Martínez & Carcavallo, 1977
	A. malheiroi	x	America	Serra, Atzingen & Serra, 1980
Bolboderini	*Belminus corredori*	x	America	Galvão & Angulo, 2006
	B. costaricencis	x	America	Herrer, Lent & Wygodzinsky, 1954
	B. ferroae	x	America	Sandoval, Pabón, Jurberg and Galvão 2007
	B. herreri	x	America	Lent & Wygodzinsky, 1979
	B. laportei	x	America	Lent, Jurberg & Carcavallo, 1995
	B. peruvianus	x	America	Herrer, Lent & Wygodzinsky, 1954
	B. pittieri	x	America	Osuna & Ayala, 1993
	B. rugulosus	x	America	Stål, 1859
	Bolbodera scabrosa	-	America	Valdés, 1910
	Microtriatoma borbai	x	America	Lent & Wygodzinsky, 1979
	M. trinidadensis	x	America	(Lent, 1951)
	Parabelminus carioca	x	America	Lent, 1943
	P. yurupucu	x	America	Lent & Wygodzinsky, 1979
Cavernicolini	*Cavernicola lenti*	x	America	Barrett & Arias, 1985
	C. pilosa	x	America	Barber, 1937
Rhodniini	*Psammolestes arthuri*	x	America	(Pinto, 1926)
	P. coreodes	x	America	Bergroth, 1911
	P. tertius	x	America	Lent & Jurberg, 1965
	Rhodnius amazonicus	x	America	Almeida, Santos & Sposina, 1973
	R. barretti	x	America	Abad-Franch, Pavan, Jaramillo, Palomeque, Dale, Chaverra & Monteiro, 2013
	R. brethesi	x	America	Matta, 1919
	R. colombiensis	x	America	Mejia, Galvão & Jurberg, 1999
	R. dalessandroi	x	America	Carcavallo & Barreto, 1976
	R. domesticus	x	America	Neiva & Pinto, 1923

	R. ecuadoriensis	x	America	Lent & León, 1958
	R. milesi	x	America	Carcavallo, Rocha, Galvão & Jurberg, 2001 (in: Valente et al. 2001)
	R. marabaensis	x	America	dos Santos Souza, Barbosa Von Atzingen, Furtado, de Oliveira, Damieli Nascimento, Pagotto Vendrami, Aristeu da Rosa 2016
	R. montenegrensis	x	America	Rosa, Rocha, Gardim, Pinto, Mendonça, Ferreira Filho, Carvalho, Camargo, Oliveira, Nascimento, Cilense, Almeida, 2012
	R. nasutus	x	America	Stål, 1859
	R. neglectus	x	America	Lent, 1954
	R. neivai	x	America	Lent, 1953
	R. pallescens	x	America	Barber, 1932
	R. paraensis	x	America	Sherlock, Guitton & Miles, 1977
	R. pictipes	x	America	Stål, 1872
	R. prolixus	x	America	Stål, 1859
	R. robustus	x	America	Larrousse, 1927
	R. stali	x	America	Lent, Jurberg & Galvão, 1993
	R. taquarussuensis	x	America	da Rosa, Garcia Justino, Nascimento, Mendonça, Rocha, Blanco de Carvalho, Falcone, Vilela de Azeredo-Oliveira, Chaboli Alevi, de Oliveira 2017
	R. zeledoni	x	America	Jurberg, Rocha, Galvão, 2009
Triatomini	*Dipetalogaster maxima*	x	America	(Uhler, 1894)
	Eratyrus cuspidatus	x	America	Stål, 1859
	E. mucronatus	x	America	Stål, 1859
	Hermanlentia matsunoi	x	America	(Fernández-Loayza, 1989)
	Linshcosteus carnifex	-	Asia	Distant, 1904
	L. chota	-	Asia	Lent & Wygodzinsky, 1979
	L. confumus	-	Asia	Ghauri, 1976
	L. costalis	-	Asia	Ghauri, 1976
	L. kali	-	Asia	Lent & Wygodzinsky, 1979
	L. karupus	-	Asia	Galvão, Patterson, Rocha & Jurberg, 2002

Mepraia gajardoi	x	America	Frias, Henry & Gonzalez, 1998
M. parapatrica	x	America	Frias-Lasserre 2010
M. spinolai	x	America	(Porter, 1934)
Panstrongylus chinai	x	America	(Del Ponte, 1929)
P. diasi	x	America	Pinto & Lent, 1946
P. geniculatus	x	America	(Latreille, 1811)
P. guentheri	x	America	Berg, 1879
P. howardi	x	America	(Neiva, 1911)
P. humeralis	x	America	(Usinger, 1939)
P. lenti	x	America	Galvão & Palma, 1968
P. lignarius	x	America	(Walker, 1873)
P. lutzi	x	America	(Neiva & Pinto, 1923)
P. martinezorum	x	America	Ayala 2009
P. megistus	x	America	(Burmeister, 1835)
P. mitarakaensis	x	America	(Bérenger & Blanchet 2007)
P. rufotuberculatus	x	America	(Champion, 1899)
P. tupynambai	x	America	Lent, 1942
Paratriatoma hirsuta	x	America	Barber, 1938
Triatoma amicitiae	-	Asia	Lent, 1951
T. arthurneivai	x	America	Lent & Martins, 1940
T. bahiensis	x	America	Sherlock & Serafim, 1967
T. baratai	x	America	Carcavallo & Jurberg, 2000
T. barberi	x	America	Usinger, 1939
T. bassolsae	x	America	(Alejandre Aguilar, Nogueda Torres, Cortéz Jimenez, Jurberg, Galvão & Carcavallo, 1999)
T. bolivari	x	America	Carcavallo, Martínez & Pelaez, 1987
T. boliviana	x	America	Martínez Avendaño, Chávez Espada,Gil, Aranda Asturizaga, Vargas Mamani, Vidaurre Prieto, 2007
T. bouvieri	-	Asia	Larrousse, 1924
T. brailovskyi	x	America	Martínez, Carcavallo & Pelaez, 1984
T. brasiliensis	x	America	Neiva, 1911

T. breyeri	x	America	Del Ponte, 1929
T. bruneri	x	America	Usinger, 1944
T. carcavalloi	x	America	Jurberg, Rocha & Lent, 1998
T. carrioni	x	America	Larrousse, 1926
T. cavernicola	-	Asia	Else & Cheong, 1977
T. circummaculata	x	America	(Stål, 1859)
T. costalimai	x	America	Verano & Galvão, 1958
T. deaneorum	x	America	Galvão, Souza & Lima, 1967
T. delpontei	x	America	Romaña & Abalos, 1947
T. dimidiata	x	America	(Latreille, 1811)
T. dispar	x	America	Lent, 1950
T. eratyrusiformis	x	America	Del Ponte, 1929
T. flavida	x	America	(Neiva, 1911)
T. garciabesi	x	America	Carcavallo, Cichero, Martínez, Prosen & Ronderos, 1967
T. gerstaeckeri	x	America	(Stål, 1859)
T. gomeznunezi	x	America	Martínez, Carcavallo & Jurberg, 1994
T. guasayana	x	America	Wygodzinsky & Abalos, 1949
T. guazu	x	America	Lent & Wygodzinsky, 1979
T. hegneri	x	America	Mazzotti, 1940
T. incrassata	x	America	Usinger, 1939
T. indictiva	x	America	Neiva, 1912
T. infestans	x	America	(Klug, 1834)
T. jatai	x	America	Gonçalves, Teves-Neves, Santos-Mallet, Carbajal de la Fuente, Lopes, 2013
T. juazeirensis	x	America	Costa y Felix, 2007
T. jurbergi	x	America	Carcavallo, Galvão & Lent, 1998
T. klugi	x	America	Carcavallo, Jurberg, Lent & Galvão, 2001
T. lecticularia	x	America	(Stål, 1859)
T. lenti	x	America	Sherlock & Serafim, 1967
T. leopoldi	-	Asia	(Schoudeten, 1933)
T. limai	x	America	Del Ponte, 1929

T. longipennis	x	America	(Usinger, 1939)
T. maculata	x	America	(Erichson, 1848)
T. matogrossensis	x	America	Leite & Barbosa, 1953
T. mazzottii	x	America	(Usinger, 1941)
T. melanica	x	America	Neiva & Lent, 1941
T. melanocephala	x	America	Neiva & Pinto, 1923
T. mexicana	x	America	(Herrich-Schaeffer, 1848)
T. migrans	-	Asia	Breddin, 1903
T. neotomae	x	America	Neiva, 1911
T. nigromaculata	x	America	(Stål, 1872)
T. nitida	x	America	Usinger, 1939
T. obscura	-	America	Maldonado & Farr, 1962
T. oliveirai	x	America	(Neiva, Pinto & Lent, 1939)
T. pallidipennis	x	America	(Stål, 1872)
T. patagonica	x	America	Del Ponte, 1929
T. peninsularis	x	America	Usinger, 1940
T. petrochiae	x	America	Pinto & Barreto, 1925
T. phyllosoma	x	America	(Burmeister, 1835)
T. picturata	x	America	(Usinger, 1939)
T. pintodiasi	x	America	Jurberg, Cunha, Cailleaux, Raigorodschi, Souza Lima, da Silva Roch, Figueiredo Moreira, 2013
T. platensis	x	America	Neiva, 1913
T. protracta	x	America	(Uhler, 1894)
T. pseudomaculata	x	America	Corrêa & Espínola, 1964
T. pugasi	-	Asia	Lent, 1953
T. recurva	x	America	(Stål, 1868)
T. rubida	x	America	(Uhler, 1894)
T. rubrofasciata	x	America/Asia	(De Geer, 1773)
T. rubrovaria	x	America	(Blanchard, 1843)
T. ryckmani	x	America	Zeledón & Ponce, 1972
T. sanguisuga	x	America	(Leconte, 1855)
T. sherlocki	x	America	Papa, Jurberg, Carcavallo, Cerqueira & Barata, 2002

	T. sinaloensis	x	America	Ryckman, 1962
	T. sinica	-	Asia	Hsiao, 1965
	T. sordida	x	America	(Stål, 1859)
	T. tibiamaculata	x	America	(Pinto, 1926)
	T. vandae	x	America	Carcavallo, Jurberg, Rocha, Galvão, Noireau & Lent, 2002
	T. venosa	x	America	(Stål, 1872)
	T. vitticeps	x	America	(Stål, 1859)
	T. williami	x	America	Galvão, Souza & Lima, 1965
	T. wygodzinskyi	x	America	Lent 1951
Fossils	*P. hispaniolae*	-	America	Poinar 2013
	T. dominicana	-	America	Poinar 2005

References

1. Justi, S. A. & Galvão, C. The Evolutionary Origin of Diversity in Chagas Disease Vectors. *Trends Parasitol.* **33,** 42–52 (2017).
